# Status of the latest 2016 World Health Organization recommended frequency of antenatal care contacts in Sierra Leone: a nationally representative survey

**DOI:** 10.1186/s12913-022-08594-y

**Published:** 2022-09-28

**Authors:** Quraish Sserwanja, Milton W. Musaba, Kassim Kamara, Linet M. Mutisya, David Mukunya

**Affiliations:** 1 Programs Department, GOAL, Khartoum, Sudan; 2 Department of Obstetrics and Gynaecology, Busitema University, Mbale, Uganda; 3 National Disease Surveillance Programme, Ministry of Health and Sanitation, Free town, Sierra Leone; 4 Maternal and Child Health Project, Swedish Organization for Global Health, Mayuge, Uganda; 5 Department of Public Health, Busitema University, Mbale, Uganda; 6 Department of Research, Nikao Medical Center, Kampala, Uganda

**Keywords:** Antenatal care, Women, Utilisation, Sierra Leone, DHS

## Abstract

**Background:**

Timely and increased frequency of quality antenatal care (ANC) contacts is one of the key strategies aimed at decreasing maternal and neonatal deaths. In 2016, the World Health Organization (WHO) revised the ANC guidelines to recommend at least eight ANC contacts instead of four. This study aimed to determine the proportion of women who received eight or more ANC contacts and associated factors in Sierra Leone.

**Methods:**

We used Sierra Leone Demographic and Health Survey (UDHS) 2019 data of 5,432 women aged 15 to 49 years who had a live birth, within three years preceding the survey. Multistage stratified sampling was used to select study participants. We conducted multivariable logistic regression to identify factors associated with utilisation of eight or more ANC contacts using SPSS version 25 complex samples package.

**Results:**

Out of 5,432 women, 2,399 (44.8%) (95% CI: 43.1–45.7) had their first ANC contact in the first trimester and 1,197 (22.0%) (95% CI: 21.2–23.4) had eight or more ANC contacts. Women who had their first ANC contact after first trimester (adjusted odds ratio, aOR, 0.58, 95% CI 0.49–0.68) and women aged 15 to 19 years had less odds of having eight or more contacts (aOR 0.64, 95% CI 0.45 to 0.91). Working (aOR 1.33, 95%CI 1.10 to 1.62) and wealthier women had higher odds of having eight or more contacts compared to poorer ones and those not working respectively. Women residing in the southern region, those using internet and less parous (less than five) women were associated with higher odds of having eight or more ANC contacts. Women who had no big problem obtaining permission to go health facilities also had higher odds of having eight or more ANC contacts compared to those who had big problems.

**Conclusion:**

Sierra Leone’s adoption of eight or more ANC contacts is low and less than half of the women initiate ANC in the first trimester. To ensure increased access to recommended ANC visits, timely ANC should be encouraged. Attributes of women empowerment such as workings status, socio-economic status, and decision-making should also be emphasized.

**Supplementary Information:**

The online version contains supplementary material available at 10.1186/s12913-022-08594-y.

## Introduction

Globally, 295,000 maternal deaths were registered in 2017 and majority of these occurred in low-income countries [1]. Sub-Saharan Africa accounts for 196, 000 (66%) of these deaths [1]. Three sub-Sahara African countries; South Sudan, Chad and Sierra Leone have the highest maternal mortality ratios globally [1]. In 2019, the maternal mortality ratio in Sierra-Leone was 717 deaths per 100,000 live births and the neonatal mortality rate was 31 deaths per 1,000 live births [2]. The third Sustainable Development Goal (SDG) targets to reduce maternal deaths to less than 70 per 100,000 live births, and the neonatal mortality to 12 per 1,000 live births or lower by 2030 [3]. To achieve this target, interventions that have been shown to reduce maternal and neonatal mortality must be scaled up [4].

Timely and increased frequency of quality Antenatal Care (ANC) contacts is one of the strategies that have been documented to decrease maternal and neonatal deaths [5–8]. Several studies have found high coverage of quality ANC contacts to be associated with reduced risk of pregnancy complications and adverse pregnancy outcomes [3, 9–12]. The reduction in maternal and neonatal mortality results from health education, timely disease prevention, screening and treatment [3, 4]. Timely ANC access also maximizes the window of opportunity for ANC skilled providers to counsel pregnant women to opt for skilled birth attendance [8, 13, 14].

The World Health Organization (WHO) previously recommended a Focused Antenatal Care (FANC) Model that emphasized at least four ANC visits [3]. In 2016, the World Health Organization (WHO) modified the minimum number of ANC visits from four to at least eight contacts, with the first contact to be made within the first 12 weeks of gestation [15–17]. “*The new WHO ANC model highlights that a woman's ‘contact’ with her provider should be more than a simple ‘visit’ but should be an opportunity for good quality care including medical care, support, and timely and relevant information throughout pregnancy”* [15]**.** By using the word ‘contact’ rather than ‘visit’, the new WHO ANC guidelines ensure a more active connection between pregnant women and their healthcare providers [18]. This further ensures that women have more contact with the care providers, which accords more opportunities for health education and preparation for childbirth [3].

Sierra Leone has some of the worst maternal and child health indicators in the world including the third highest maternal mortality ratio (MMR) [1, 19, 20]. Maternal deaths account for 9% of all deaths among women of reproductive age with women having a maternal mortality lifetime risk of 3·4% [21]. Despite improvements in utilization of some maternal child health (MCH) services such as skilled birth attendance (SBA) (94% in urban areas and 84% in rural areas) [22], ANC coverage of four or more ANC contacts sluggishly increased from 76% in 2013 to 79% in 2019 while early initiation in the first trimester declined from 45% in 2013 to 44% in 2019 [2]. Several studies have identified poor quality of MCH services provided in Sierra Leon as a possible explanation for sluggishness in uptake of MCH services by communities. For instance, Koroma et al. analyzed data from 97 health facilities and three hospitals and showed quality of ANC to be poor with less than a third (27%) of women being examined, less than a half (47%) were able to receive the recommended interventions and gross noncompliance to national standards of care [23]. However, in 2018, Sierra Leone joined the Global Quality of Care Network with an aim of ensuring that every pregnant woman, newborn and child receives quality care and hence have improved maternal and child health outcomes [24]. The Sierra Leone Ministry of Health adopted the new WHO ANC model in 2017 as indicated in the Sierra Leone National Reproductive, Maternal, Neonatal, Child and Adolescent Health (RMNCAH) strategy (2017–2021) [25]. This strategy further sets targets of reducing MMR to 650 per 100,000 live births and neonatal mortality to 23 per 1,000 live births [25].

To ensure reduction in maternal and neonatal mortality, Sierra Leone needs to ensure effective implementation of the new WHO ANC model. However, there is a dearth of information on the utilization of ANC as per the latest WHO guidelines, which impedes effective implementation. The most recent Sierra Leone Demographic and Health Survey (SLDHS) provided ANC utilisation statistics using the old WHO recommendations. It is important to look deeper into the current status of the timeliness and frequency of ANC uptake to inform and guide national implementation programmes. Hence our study aimed at determining the proportion of women who received timely initiation and utilisation of eight or more ANC contacts and associated factors in Sierra Leone.

## Methods

### Data source

This study used secondary data from the 2019 SLDHS. Data were accessed from MEASURE DHS database at http://dhsprogram.com/data/available-datasets.cfm. SLDHS was a nationally representative cross-sectional survey implemented by Statistics Sierra Leone (Stats SL) with technical assistance from ICF intern through the DHS Program and funded by the United States Agency for International Development (USAID). The Demographic and Health Survey datasets are freely available to the public though researchers must register with MEASURE DHS and submit a request before accessing them.

### Study sampling and participants

The 2016 SLDHS samples were selected using a stratified, two-stage cluster sampling design that resulted in the random selection of 13,872 households [2]. The primary sampling unit (PSU), referred to as a cluster was based on enumeration areas (EAs) from the 2015 EA population census frame [2]. Stratification was achieved by separating districts into urban and rural areas with a total of 31 sampling strata created. In the first stage, 578 EAs were selected with probability proportional to EA size which was the number of households with in the EA [2]. Detailed sampling procedures were published in the final report [2]. DHS uses different questionnaires. Household questionnaire collects data on household environment, assets and basic demographic information of household members while women’s questionnaire collects data about women’s reproductive health, domestic violence and nutrition indicators. This secondary analysis included women aged 15 to 49 years who had a live birth within three years preceding the survey and were either permanent residents or slept in the selected household the night preceding the survey. Out of the total weighted sample of 15,574 women in the data set, only 7,326 and 5,432 had given birth within five and three years preceding the survey respectively**.** Of the 5,432 women that had a live birth within three years preceding the survey, 82 women had missing data on the timing of ANC first contact leading to a total of 5,350 women for logistic regression analyses**.** We chose women who had given birth within three years preceding the survey because the WHO new ANC guidelines were introduced in November 2016 [15] and the SLDHS was carried out in May 2019.

### Variables

#### Dependent variable

The outcome variable was the total number of ANC contacts. These were categorized into dichotomous variables: total number of ANC contacts (less than 8 contacts as inadequate and coded as 0 and 8 contacts and above as adequate and coded as 1). Similar analysis was done with timing of ANC initiation as the outcome (initiation within the first trimester as early initiation coded as 1 and initiation after first trimester as delayed initiation coded as 0) as shown in supplementary file 1.

#### Independent variables

This study included determinants of ANC frequency based on evidence from available literature and data [3, 13, 26–28]. Nineteen explanatory variables were used: (1) maternal age, (2) wealth index, (3) level of education, (4) place of residence, (5) region, (6) marital status, (7) working status, (8) ANC timing of first contact, (9) sex of household head, (10) household size, (11) woman’s religion, (12) parity, (13) exposure to newspapers, (14) exposure to television (TV), (15) exposure to radio, (16) internet use, (17) having problems with getting permission to seek help, (18) having problems with distance to the nearby health facility and (19) being visited by a fieldworker. Maternal age was categorised as; (15–19 years, 20–34 years and 35–49 years). Wealth index is a measure of relative household economic status and was calculated by UDHS from information on household asset ownership using Principal Component Analysis, which was further categorised into poorest, poorer, middle, richer and richest quintiles [29]. Place of Residence was categorised into urban and rural. Region was categorised into four; Northern, Eastern, Southern, Western and Northwestern while level of Education was categorised into no education, primary education, secondary and tertiary education. Household Size was categorised as less than seven members and seven and above members (based on the dataset average of seven members per household). Sex of household head was categorised as male or female, working status categorized as: not working and working while marital status as married (this included those in formal and informal unions) and not married. Religion was categorised as Muslims and Christians and others, problems seeking permission and distance to health facility were categorised as big problem and no big problem while exposure to mass media and internet use (TV, radio, and newspapers) were categorised as yes and no. In the questionnaire, seeking permission to access healthcare and distance to health facility had three original responses: no problem, no big problem and big problem. However, none of the study participants reported no problem hence we only had two responses.

### Statistical analysis

In order to account for the multi-stage cluster study design, we used SPSS version 25.0 statistical software complex samples package incorporating the following variables in the analysis plan to account for the multistage sample design inherent in the DHS dataset: individual sample weight, sample strata for sampling errors/design, and cluster number [29, 30]. Analysis was carried out based on the weighted count to account for the unequal probability sampling in different strata and to ensure representativeness of the survey results at the national and regional level.

Before logistic regression, each exposure/predictor (independent variable) was assessed separately for its association with the outcome variable using bivariable logistic regression and we presented the crude odds ratio (COR), 95% confidence interval (CI) and p-values. Independent variables associated with frequency of ANC from literature and those with a p-value ≤ 0.25 at the bi-variable level, and not strongly collinear with other independent variables were included in the final multivariable logistic regression model to assess the independent effect of each variable on the timing and frequency of ANC. Multi-collinearity was assessed using variance inflation factor (VIF) and no VIF was above 3. Adjusted odds ratios (AOR), 95% confidence intervals (CI) and p-values were calculated with statistical significance level set at *p*-value < 0.05.

## Results

A total of 5,432 women were included in the analysis (Table 1). Of these, 2,399 (44.8%) (95% CI: 43.1–45.7) had their first ANC contact in the first trimester and 1,197 (22.0%) (95% CI: 21.2–23.4) had eight or more ANC contacts. Majority of the women were residing in rural areas (64.0%), were Muslims (79.5%), had no education (52.2%), resided in male headed households (76.3%), were married (83.1%), working (75.2%) and aged between 20 and 34 years (67.6%). Mass media exposure was limited with only 41.0% of women being exposed to radio, 22.6% to TV, 9.0% using internet and 5.2% exposed to newspapers.

**Table 1:** Socio-demographic characteristics of women in Sierra Leone as per the 2019 SLDHS

Characteristics	Births in the last 3 years *N* = 5,432	%
**Age**		
15 to 19	537	9.9
20 to 34	3674	67.6
35 to 49	1221	22.5
**Visited by fieldworker**		
No	3769	69.4
Yes	1663	30.6
**Residence**		
Urban	1954	36.0
Rural	3477	64.0
**Region**		
Western	1022	18.8
Eastern	1161	21.4
Northwestern	1050	19.3
Northern	1053	19.4
Southern	1146	21.1
**Religion**		
Islam	4318	79.5
Christianity and others	1114	20.5
**Sex household head**		
Male	4143	76.3
Female	1289	23.7
**Household Size**		
7 and above	2513	46.3
Less than 7	2919	53.7
**Working status**		
Not working	1346	24.8
Working	4086	75.2
**Marital status**		
Not married	918	16.9
Married	4514	83.1
**Education Level**		
No Education	2836	52.2
Primary Education	803	14.8
Secondary Education	1647	30.3
Tertiary	145	2.7
**Wealth Index**		
Poorest	1220	22.5
Poorer	1189	21.9
Middle	1111	20.5
Richer	1036	19.1
Richest	875	16.1
**Parity**		
1	1486	27.4
2–4	3002	55.3
5 and above	944	17.4
**Exposure to newspapers**		
No	5151	94.8
Yes	281	5.2
**Exposure to Radio**		
No	3206	59.0
Yes	2226	41.0
**Exposure to TV**		
No	4206	77.4
Yes	1226	22.6
**Internet use**		
No	4944	91.0
Yes	488	9.0
**Permission to access healthcare**		
Big problem	1407	25.9
Not big problem	4025	74.1
**Distance to health facility**		
Big problem	2617	48.2
Not big problem	2815	51.8
**ANC timing** ^a^		
First trimester	2399	44.8
After first trimester	2952	55.2
**ANC attendance**		
8 contacts and above	1197	22.0
Less than 8 contacts	4235	78.0

^a^missing 82 (1.5%) respondent

### Factors associated with having eight or more ANC contacts

Women who had their first ANC contact after first trimester (adjusted odds ratio, aOR, 0.58, 95% CI 0.49–0.68) and women aged 15 to 19 years had a less likelihood of having eight or more contacts (aOR 0.64, 95% CI 0.45 to 0.91) compared to those who initiated ANC within the first trimester and older women aged 35 to 49 years respectively as shown in Table 2. Working (aOR 1.33, 95%CI 1.10 to 1.62) and wealthier women had higher odds of having eight or more contacts compared to poorer ones and those not working respectively. Women residing in the Southern region (aOR 1.64, 95%CI 1.07 to 2.52), those using internet (aOR 1.97, 95%CI 1.45 to 2.67) and less parous (para 1) women (aOR 1.36, 95%CI 1.01 to 1.85) were associated with more odds of having eight or more ANC contacts compared to women in the Western region, those not using internet and para 5 and above women respectively. Women who had no big problem obtaining permission to go health facilities (aOR 1.39, 95%CI 1.07 to 1.81) also had higher odds of having eight or more ANC contacts compared to those who had big problems.

**Table 2:** Factors associated with ANC frequency in Sierra Leone as per the 2019 SLDHS

	**ANC frequency *N* = 5,350**		
**Characteristics**	**Crude model cOR (95% CI)**	***P*-value**	**Adjusted model aOR (95% CI)**
**Age**			
35 to 49	1		1
20 to 34	0.84 (0.72–0.99)	0.045	0.71 (0.59–0.87)^*^
15 to 19	0.72 (0.53–0.98)	0.035	0.64 (0.45–0.91)^**^
**Residence**			
Rural	1		1
Urban	1.03 (0.82–1.29)	0.789	1.03 (0.71–1.50)
**Region**			
Western	1		1
Southern	1.42 (1.01–1.98)	0.042	1.64 (1.07–2.52)^*^
Northwestern	1.34 (0.93–1.94)	0.112	1.56 (0.99–2.47)
Northern	0.85 (0.58–1.25)	0.417	0.89 (0.56–1.40)
Eastern	1.25 (0.86–1.82)	0.235	1.34 (0.85–2.10)
**Religion**			
Islam	1		1
Christianity and others	1.07 (0.90–1.28)	0.279	1.04 (0.84–1.31)
**Sex household head**			
Male	1		1
Female	1.01 (0.85–1.20)	0.059	1.16 (0.95–1.42)
**Household Size**			
7 and above	1		1
Less than 7	1.10 (0.94–1.28)	0.244	1.13 (0.95–1.34)
**Working status**			
Not working	1		1
Working	1.23 (1.03–1.48)	0.024	1.33 (1.10–1.62)^**^
**Marital status**			
Not married	1		1
Married	0.96 (0.79–1.18)	0.719	0.98 (0.78–1.23)
**Education Level**			
No Education	1		1
Primary Education	1.15 (0.94–1.40)	0.176	1.15 (0.93–1.42)
Secondary Education	1.01 (0.85–1.21)	0.890	0.94 (0.76–1.16)
Tertiary	1.81 (1.21–2.69)	0.004	1.28 (0.77–2.11)
**Wealth Index**			
Poorest	1		1
Poorer	1.22 (0.98–1.51)	0.076	1.27 (1.02–1.59)^*^
Middle	1.30 (1.03–1.64)	0.025	1.39 (1.09–1.77)^**^
Richer	1.48 (1.13–1.93)	0.005	1.74 (1.23–2.46)^**^
Richest	1.06 (0.77–1.46)	0.721	1.32 (0.83–2.12)
**Parity**			
5 and above	1		1
2–4	1.15 (0.95–1.40)	0.146	1.35 (1.07–1.69)^*^
1	1.07 (0.84–1.37)	0.562	1.36 (1.01–1.85)^*^
**Newspapers exposure**			
No	1		1
Yes	1.06 (0.76–1.47)	0.749	0.90 (0.62–1.31)
**Exposure to Radio**			
No	1		1
Yes	1.08 (0.91–1.29)	0.367	1.06 (0.87–1.28)
**Exposure to TV**			
No	1		1
Yes	0.87 (0.69–1.09)	0.216	0.78 (0.61–1.00)
**Internet use**			
No	1		1
Yes	1.60 (1.22–2.10)	0.001	1.97 (1.45–2.67)^***^
**Permission to access healthcare**			
Big problem	1		1
Not big problem	1.26 (1.01–1.58)	0.041	1.39 (1.07–1.81)^*^
**Distance to health facility**			
Big problem	1		1
Not big problem	0.89 (0.74–1.07)	0.217	0.82 (0.67–1.00)
**ANC timing** ^a^			
First trimester	1		1
After first trimester	0.58 (0.50–0.68)	< 0.001	0.58 (0.49–0.68)^***^
**Visited by fieldworker**			
No			
Yes	0.96 (0.79–1.17)	0.686	0.93 (0.76–1.13)

^a^missing 113 (1.5%) respondents

^*^Significant at *p*-value < 0.05

^*^^*^Significant at *p*-value < 0.01

^***^Significant at *p*-value < 0.001, *aOR* Adjusted odds ratio, *cOR* Crude Odds Ratio. Non bolded variables in adjusted model had p-values greater than 5 and were not found to be statistically significant

Supplementary file 1 shows that women from the Southern, Northern and Eastern regions and those with no big problems getting permission to access care had higher odds of timely initiation of ANC contacts compared to those from the Western region and those with big problems getting permission to access care respectively.

## Discussion

Our study has revealed that the utilisation of eight or more ANC contacts in Sierra Leone was low (22%). Furthermore, timely attendance of the first ANC visit was also low. A couple of reasons could explain this finding: firstly, at the time of the survey, the change in ANC policy had just happened in Sierra Leone and the roll out was not complete. Secondly, the existence of structural challenges such as poor roads and transportation networks to health facilities could explain the poor utilization of the eight or more contacts [31]. Our findings are similar to those reported by a study that used Nigeria DHS data at 20.3% [32], but higher than what was reported in Benin (8%) [33], the pooled prevalence of 7.7% from DHS data of eight countries in SSA (Guinea 3.3%, Mali 3.5%, The Gambia 4.3%) [32], 6.8% from 36 SSA countries [34], and 13.0% DHS data of Asian and SSA countries [35]. As much as this shows that Sierra Leone has one of the highest proportions of utilization of eight or more ANC contacts compared to other countries in SSA, is still below the level recommended by WHO [15]. Although less than half of the women initiated ANC in the first trimester, it is higher compared to a study conducted in Northern Bangladesh (14%) [3], Kenya 10.9% [36] but lower than that in Doula, Cameroon (56%) [28]. These differences in the observed prevalence could be partially explained by variations in the time, study settings can partially explain the differences. Studies that have reported lower prevalence were done earlier than our study while maternal health indicators have been shown to improve with time. The study in Cameroon that showed a higher proportion of women initiating ANC in the first trimester was conducted in a referral hospital located in the economic capital of Cameroon, while our study utilized a nationally representative sample combining both rural and urban areas. To the best of our knowledge, this study is the foremost to explore the status and extent of in-country utilisation of ANC as per the new WHO guidelines of 2016.

Our results further reveal that women in the southern region, who were working, of low parity, accessed and used internet and had no big problem seeking permission to access healthcare had higher odds of utilising at least 8 ANC contacts compared to those in the Western region who were not working, had higher parity, had no access to internet and had big problems seeking permission to access healthcare respectively. Furthermore, women who were younger and those who initiated ANC after the first trimester had lower odds of utilising at least eight (8) ANC contacts compared to their counterparts who were older and those initiated ANC contacts in the first trimester. While the guidelines further recommend that initiation of the first ANC contact should occur in the first trimester, less than half (44.8%) of the women-initiated ANC in the first trimester.

Belonging to a higher wealth index was associated with higher odds of utilising 8 or more ANC contacts. This is similar to other studies conducted using SSA multi-country DHS data [32, 34, 35]. Financial constraints, limited access to health facilities, limited decision-making power in regard to reproductive health matters have been linked to poor utilisation of ANC services [33]. Wealth index, a proxy of financial status may indicate that wealthier women have enough funds which enable them afford direct and indirect costs involved in accessing quality and timely healthcare services [33, 37]. Furthermore, women from wealthier households are more enlightened and empowered hence have more decision making powers which enables them access healthcare more frequently and timely [14, 33]. Given that Sierra Leone has free maternal healthcare services [38], our results suggest that, apart from the direct costs of health services, indirect costs may play a key role in influencing ANC utilisation. This is consistent with findings from other studies that reported economic factors such as transportation costs and miscellaneous fees paid for healthcare to influence the women’s utilisation of maternal healthcare [39, 40]. Therefore, there is need for further research to explore the influence of indirect costs on accessing and utilizing ANC.

Region has been shown to influence the frequency of ANC utilisation. Unexpectedly, women in the southern region were associated with higher odds of utilising eight or more ANC contacts compared to women in the western region. In Sierra Leone, the Western region has the largest concentration of health workers, it is the most developed and houses the capital and is the economic city of the country and hence has higher quality social amenities compared to other regions [38, 41]. However, the increasing numbers of urban poor in the developed Western region coupled with high standards of living and inequitable distribution of social amenities including public and private health facilities, make it hard for low income women to access the services. Furthermore, the documented staff challenges such as poor delegation, favoritism and a lack of autonomy could partly affect quality of services in public health facilities which further limits access to these facilities by pregnant women [38, 41]. The efforts of the government to ensure better service delivery in other regions that are far away from the capital could also have contributed to this observation [25]. However, more studies are needed to explore these regional differences in the utilisation of ANC. The role of regional disparities in explaining ANC utilisation has also been documented in previous studies in Uganda and Nigeria [14, 26, 42].

Younger age was associated with lower odds of utilising 8 or more ANC contacts. Mixed results have been documented in previous studies on the association between the pregnant woman’s age and utilisation of ANC contacts. A study in Northwestern Ethiopia showed younger age to be associated with better ANC utilisation compared to older age [43] which was a similar finding by a study that analysed 2011 Ethiopia DHS data and showed older women to be more likely to have less ANC utilisation [27]. Similar to our findings, studies in Ghana, Tanzania and Zambia showed younger age to be associated with lower odds of increased ANC utilisation [44–46]. Furthermore, a study that analyzed SSA DHS pooled data and showed results similar to those in this study [32, 35]. Younger age has been associated with limited knowledge on the importance of ANC, they have higher odds of unplanned and unwanted pregnancies which limits access to maternal care even to the extent of not utilising ANC contacts at all [27, 44]. Younger age is also associated with limited decision making power and financial constraints which further limit access to maternal care [45]. Furthermore, limited social support from family and adolescent unfriendly services for young pregnant women might also partly explain the limited utilization of ANC by young women. A study that explored the causes of maternal mortality among teenagers in Sierra Leone and showed pregnant adolescents are commonly rejected by their families, some experience physical abuse from their fathers and some are mocked at by healthcare providers [47]. Some of these young women who are forced to run away from home to stay with peers who also inexperienced with maternal care usually with no reliable source of support [47]. All this stigma, abandonment, and limited family-based support negatively affects health seeking behaviour hence delayed or no care-seeking for antenatal and delivery care [47]. A recent assessment shows that only 33.3% of the sampled health facilities had available space that can be converted to provide adolescent and young people friendly services (AYFHS), only 66.7% had at least one staff trained in providing AYFHS training and only a third were using peer educators [48]. Given the high prevalence of teenage pregnancy in Sierra Leone [2], this finding reinforces the need to intensify advocacy messages aimed at promoting increased ANC utilisation among younger women and prioritizing pregnant adolescents in health facilities to ensure that they receive adolescent friendly services. However, beyond the scope of our study, it would be interesting to further explore the underlying factors mediating the effect of age of mother on utilisation of ANC.

Exposure to TV, radio and newspapers were not associated with ANC utilisation while using internet was associated with higher odds of utilising eight or more ANC contacts. Exposure to media has been shown in previous studies to have a positive association with ANC utilisation [3, 8, 49–51]. Absence of association between exposure to newspaper or magazines and ANC utilisation could partly be attributed to the low levels of education with over 52.7% having no education and the fact that most women reside in rural areas where access to Newspapers or magazines is hard and not sustainable due to the daily or weekly costs involved. Internet use was associated with higher odds of increased ANC utilisation. Different internet resources such as web pages, social media platforms, bulletin boards, and chatrooms may contain health information and provide access to information [52, 53]. Access to this information on the internet helps in reducing knowledge gaps by sensitising women on the benefits of ANC utilisation which leads to positive attitudes, challenges negative social norms and improves health seeking behavior [49, 54].

Our findings also reveal that working women had higher odds of utilising eight or more ANC contacts compared to those who were not working. Women who work are more likely have better financial status which enables them to afford the direct and indirect costs associated with access to maternal healthcare [34]. The importance of working status in influencing ANC utilisation has been documented in the previous literature [26, 34, 55].

The association between parity and ANC utilisation, with those of low parity having more ANC contacts compared to the multiparous (5 and above) women. This has been observed in similar studies [26, 44, 55]. Multiparous women tend to have a false sense of perceived low risk due to prior experience of [44, 55]. Furthermore, limited time availability due to the high the responsibilities of other children might make it hard for these women to access 8 or more ANC contacts [56, 57]. In addition, women who initiated ANC contacts after the first trimester had lower odds of attending 8 or more ANC contacts. Similarly, the findings from previous studies showed that early booking for ANC would result in optimal number of ANC contacts during pregnancy [33, 58]. Timely initiation of ANC contacts ensures that the women get timely and adequate health education sessions regarding the benefits of maternal care during pregnancy and further creates rapport between the health workers and the pregnant women [8, 59]. It’s also possible that those who initiate ANC in the first trimester have better health seeking behaviour and hence are more likely to utilise more ANC contacts.

Problems in seeking permission to access healthcare decision was another variable found to be associated with timing and frequency of ANC contacts. Women who had less problems in seeking permission were more likely to initiate ANC early and utilise 8 or more ANC contacts compared to those who had more problems. This may be a depiction of low women’s empowerment and cultural beliefs. Studies have emphasised the importance of improving women’s empowerment mainly in the aspect of healthcare seeking and decision-making [27, 60]. In addition, creation of awareness through mass media to provide information about the importance of empowering women so as to seek healthcare freely would be helpful in facilitating behavior change and ensuring increased ANC utilisation [26, 33]. Several studies have documented the effect of low decision-making powers of women in accessing and seeking ANC [27, 33, 43].

Surprisingly, higher education was not associated with increased odds of having attained 8 or more ANC contacts. This is confusing, as other studies have shown an association between higher education and more ANC contacts [50].

### Strengths and limitation

This study used the most recent SLDHS data with a larger sample size and higher quality, which substantially reduces the risk of sampling bias and measurement bias. The findings of the study also provide timely evidence for maternal healthcare stakeholders and policy-makers with respect to effective implementation of the 2016 WHO ANC model and ensuring reduction of maternal and infant mortality, which highly depend on increased use of reproductive and maternal health services. This study used cross-sectional data which only enables associations to be established but not causal relationships. The other limitation was that the SLDHS did not include information on crucial factors such as male involvement in ANC, knowledge of ANC contacts in the context of timing and frequency, timing of the subsequent ANC contacts and the quality of healthcare which could have an effect on uptake of ANC services.

## Conclusion

Less than a third (22%) of women in Sierra Leone attended eight or more ANC contacts. This study revealed that Western region, poor, younger women and those who are not working, who don’t use internet, initiate ANC late, multiparous and having problems seeking permission to seek healthcare had lower odds of adequate ANC utilisation. Generally, there is need for maternal healthcare stakeholders to prioritize young, poor, multiparous women from the Western region. There is need to leverage the enormous potential of internet by making it more accessible and affordable and hence be used to access information on maternal healthcare. Providing safe and friendly spaces and healthcare in health facilities for young pregnant women needs to be further strengthened to motivate adolescent and young women access care with ease. ANC providers need to strengthen follow up mechanisms mainly among multiparous women to increase their utilization of follow up visits after the first visit.

## Supplementary Information

**Additional file 1.** 

## Data Availability

The data set used is openly available upon permission from MEASURE DHS website (URL: https://www.dhsprogram.com/data/available-datasets.cfm).

## Abbreviations

EA: Enumeration area
AOR: Adjusted Odds Ratio
CI: Confidence Interval
COR: Crude Odds Ratio
DHS: Demographic Health Survey
SLDHS: Sierra Leone Demographic Health Survey
OR: Odds Ratio
SD: Standard Deviation
WHO: World Health Organization
ANC: Antenatal care
SPSS: Statistical Package for Social Science

## Declarations

### Availability of data and materials

The data set used in this study is openly available from MEASURE DHS website and the modified data set that was used for the final analysis can be availed upon request from the corresponding author.
