# Supplementary material for: Status of the latest 2016 World Health Organization recommended frequency of antenatal care contacts in Sierra Leone: a nationally representative survey
Source: BMC Health Serv Res. 2022 Sep 28;22:1208. doi: 10.1186/s12913-022-08594-y (PMC9520872; doi:10.1186/s12913-022-08594-y)
Supplement: Supplementary file 1 — Additional file 1. [file 12913_2022_8594_MOESM1_ESM.docx]

**Factors associated with timing of ANC initiation in Sierra Leone as per the 2019 SLDHS**

|  | **ANC timing**  **N=5,350** | | |
| --- | --- | --- | --- |
| **Characteristics** | **Crude model**  **cOR (95% CI)** | **P-value** | **Adjusted model**  **aOR (95% CI)** |
| **Age** |  |  |  |
| 35 to 49 | 1 |  | 1 |
| 20 to 34 | 1.08 (0.93-1.26) | 0.319 | 1.11 (0.94-1.32) |
| 15 to 19 | 0.96 (0.77-1.18) | 0.678 | 0.97 (0.73-1.29) |
| **Residence** |  |  |  |
| Rural | 1 |  | 1 |
| Urban | 0.90 (0.76-1.07) | 0.242 | 0.91 (0.71-1.16) |
| **Region** |  |  |  |
| Western | 1 |  | 1 |
| Southern | 1.29 (0.99-1.69) | 0.060 | **1.37 (1.01-1.88)*** |
| Northwestern | 0.91 (0.68-1.21) | 0.524 | 0.93 (0.66-1.32) |
| Northern | 1.47 (1.10-1.95) | 0.008 | **1.51 (1.10-2.08)*** |
| Eastern | 2.06 (1.52-2.78) | <0.001 | **2.23 (1.58-3.15)***** |
| **Religion** |  |  |  |
| Islam | 1 |  | 1 |
| Christianity and others | 1.16 (0.98-1.38) | 0.079 | 1.02 (0.88-1.18) |
| **Sex household head** |  |  |  |
| Male | 1 |  | 1 |
| Female | 0.96 (0.82-1.12) | 0.582 | 0.96 (0.83-1.11) |
| **Household Size** |  |  |  |
| 7 and above | 1 |  | 1 |
| Less than 7 | 1.05 (0.93-1.19) | 0.438 | 1.01 (0.89-1.15) |
| **Working status** |  |  |  |
| Not working | 1 |  | 1 |
| Working | 0.99 (0.84-1.17) | 0.910 | 0.94 (0.79-1.11) |
| **Marital status** |  |  |  |
| Not married | 1 |  | 1 |
| Married | 1.07 (0.91-1.27) | 0.424 | 1.11 (0.91-1.36) |
| **Education Level** |  |  |  |
| No Education | 1 |  | 1 |
| Primary Education | 1.14 (0.94-1.39) | 0.174 | 1.12 (0.92-1.37) |
| Secondary Education | 0.98 (0.84-1.15) | 0.806 | 0.95 (0.79-1.14) |
| Tertiary | 1.24 (0.82-1.87) | 0.313 | 1.26 (0.79-2.00) |
| **Wealth Index** |  |  |  |
| Poorest | 1 |  | 1 |
| Poorer | 1.07 (0.89-1.29) | 0.470 | 1.12 (0.92-1.36) |
| Middle | 1.04 (0.86-1.28) | 0.670 | 1.12 (0.91-1.38) |
| Richer | 1.02 (0.82-1.28) | 0.829 | 1.25 (0.96-1.63) |
| Richest | 0.88 (0.67-1.15) | 0.337 | 1.14 (0.79-1.66) |
| **Parity** |  | 0.715 |  |
| 5 and above | 1 |  | 1 |
| 2-4 | 0.98 (0.85-1.14) | 0.804 | 0.93 (0.79-1.10) |
| 1 | 0.98 (0.81-1.18) | 0.163 | 1.01 (0.78-1.29) |
| **Newspapers exposure** |  |  |  |
| No | 1 |  | 1 |
| Yes | 0.98 (0.72-1.32) | 0.875 | 1.00 (0.71-1.40) |
| **Exposure to Radio** |  |  |  |
| No | 1 |  | 1 |
| Yes | 1.09 (0.95-1.26) | 0.218 | 1.12 (0.96-1.30) |
| **Exposure to TV** |  |  |  |
| No | 1 |  | 1 |
| Yes | 0.94 (0.80-1.12) | 0.512 | 1.00 (0.80-1.25) |
| **Internet use** |  |  |  |
| No | 1 |  | 1 |
| Yes | 0.96 (0.73-1.27) | 0.790 | 1.00 (0.70-1.44) |
| **Permission to access healthcare** |  |  |  |
| Big problem | 1 |  | 1 |
| Not big problem | 1.10 (0.92-1.32) | 0.308 | **1.27 (1.05-1.54)*** |
| **Distance to health facility** |  |  |  |
| Big problem | 1 |  | 1 |
| Not big problem | 0.93 (0.80-1.09) | 0.394 | 0.90 (0.76-1.06) |
| **Visited by fieldworker** |  |  |  |
| No | 1 |  |  |
| Yes | 1.01 (0.87-1.18) | 0.880 | 0.97 (0.83-1.12) |

^a^_=_ missing 113 (1.5%) respondents. ***=** Significant at p-value <0.05, ****=** Significant at p-value <0.01, *****=** Significant at p-value <0.001, aOR: Adjusted odds ratio. cOR: Crude Odds Ratio
